# Abstracts from the Royal College of Podiatry Annual Conference: Liverpool, UK 18 – 21 November 2021www.rcop.org.uk

**DOI:** 10.1186/s13047-022-00529-4

**Published:** 2022-05-10

## ORAL PRESENTATIONS

### Jewel in the Crown

#### JIC.1 Patients' perspectives on satisfaction and comparison between online video and face-to-face consultations in a private musculoskeletal clinic: a cross-sectional questionnaire study

##### Alice Corbett

###### Queen Mary University, London, UK

####### **Correspondence:** Alice Corbett

**Background:** The COVID-19 pandemic and lockdown restrictions drastically modified the delivery of clinical care in musculoskeletal (MSK) settings. Providers rapidly replaced traditional face-to-face consultation (F2FC) with video consultations (VC), requiring patients to quickly adapt to this new modality. There is uncertainty regarding how patients responded to VCs and the influence upon their assessment. This study aimed to identify and evaluate participant satisfaction levels and preference for modality following both a VC and F2FC.

**Methods:** A cross-sectional questionnaire study was conducted for patients who had experienced both modalities under a private London-based MSK clinic. A 35-item questionnaire was developed specifically for the purpose of this study. Quantitative and qualitative items were used to ensure a wide range of feedback was achieved. Following a pilot study, the online questionnaire was launched and distributed to participants via email.

**Results:** 177 of 500 (35.4%) invited participants completed the questionnaire. Results identified a statistically significant higher level of participant satisfaction with F2FC. It was perceived as superior to VC in the majority of clinical applications and the preference for future appointments by 86.4% of participants.

**Conclusions:** Traditional physical interactions and communication were the leading factors of satisfaction and preference for F2FC, which were perceived as limitations with VC. Participants identified VC as a valuable tool and recommended it to be used in conjunction with F2FC. Participants reported higher satisfaction levels and a stronger preference for F2FC for their MSK complaint, but still supported the implementation of VC as a future option.

### A7

#### A7.2 Towards a better risk stratification model for people with diabetes at risk of incident foot ulceration: a prognostic modelling analysis of patients on the SCI-Diabetes register

##### Joanne Hurst^1^; Ruth Barn^1^; Hamish Innes^1^; Sicco Bus^2^; Brian Kennon^3^; James Woodburn^1^

###### ^1^School of Health and Life Sciences, Glasgow Caledonian University, Glasgow, UK; ^2^Academisch Medisch Centrum, Universiteit van Amsterdam, The Netherlands; ^3^Department of Podiatry, Queen Elizabeth University Hospital, Glasgow, UK

####### **Correspondence:** Joanne Hurst

**Background:** The aim of the study was to assess if adding information on clinical, systemic and social environmental factors to the Scottish Foot Ulcer Risk Score (SFURS) improves risk stratification for diabetic foot ulceration.

**Method:** We extracted and analysed the anonymised digital health data from 59,582 people with diabetes from the Scottish Care Information – Diabetes Collaboration (SCI-diabetes) register. We extracted data on their earliest SFURS score and supplemented this with information on age, gender, time since diabetes diagnosis, Charlson comorbidity index, Scottish Index of Multiple deprivation, ethnicity and type of diabetes. We recorded incident diabetic foot ulcer event between 2007-2016. Using survival analysis methods, we compared the discriminative performance of the following two predictive models using Harrel’s C-statistic: (MODEL-1) existing SFURS score; and (MODEL-2) SFURS plus all the aforementioned prognostic factors.

**Results:** Over a 9.8-year period, incident DFU was observed in 2235 (3.8%) registry patients. Cox regression modelling indicated that a high risk SFURS score was the strongest risk factor of foot ulceration (Adjusted hazard ratio (HR) 8.24 [95%CI 7.27-9.33], *P*= <0.001).

**Conclusions**: Other factors that were independently associated with foot ulceration were increased disease duration (adjusted HR for 16+ years vs 0-5 years: 3.22 [95%CI 2.89-3.70] *P*= <0.001), being male (adjusted HR vs females: 1.40 [95% CI 1.28-1.54], P= <0.001), and increased exposure to social deprivation (adjusted HR for least deprived vs most deprived quintile: 0.83 [95% 0.72-0.96] *P*= 0.012). The Harrel’s C statistic was superior for MODEL-2 (C-stat:0.75) versus MODEL-1(C-stat: 0.68), indicating that the broader model was better at differentiating individuals who go onto develop a DFU from those who do not.

#### A7.3 Staggered Steps - The impact of multiple sclerosis on lower limb health

##### Ailsa Baumgartner

###### Discipline of Podiatric Medicine, National University of Ireland, Galway, Ireland

####### **Correspondence:** Ailsa Baumgartner

**Background:** Multiple Sclerosis (MS) is a complex disease which affects the central nervous system. The myelin sheath that protects the nerve fibres of the body is damaged by its own immune system (demyelination). Under NICE guidelines CG186, podiatrists are not currently included within the Multiple Sclerosis rehabilitation team, with access to podiatric treatment in secondary care available for those with a specific need.

**Methods:** A scoping review of the effects of MS on the lower limb (using PRISMA-ScR) was undertaken to examine the extent, range and nature of any research activity that has already been completed in this area of podiatric medicine, subsequently identifying research gaps in existing literature. A PPI advisory panel was established, comprising of ten members, aimed at highlighting the effects of MS on lower limb health. The PPI advisory panel was formed through social media recruitment, utilising Twitter as the primary platform.

**Results:** The COM-B model has been used as a framework for analysis, along with keywords used as discussion strategy. The Guidance for Reporting Involvement of Patients and Public 2 - GRIPP2 is the checklist that has been utilised to report the themes and initial findings of the PPI meetings. This guidance resembles the logic model, highlighting that evaluating participation is a complex activity, which provides the fundamental key to ensuring that public involvement and participation activities and programmes generate learning and results, and, improve future participation practices.

**Conclusions:** The initial findings have identified that there is scope for further research of the utilisation of podiatrists in the management and rehabilitation of lower limb health in patients with MS. The dialogue of the panel is encouraging with a host of examples provided as evidence that podiatric interventions could facilitate meaningful change to the rehabilitation service and have a great impact on both patients and carers.

#### A7.4 Screening for Depressive Symptoms in Patients with Diabetic Peripheral Neuropathy

##### Cynthia Formosa, Alfred Gatt; Rahab Marhoon Alghafri

###### Faculty of Health Sciences, University of Malta, Msida, Malta

####### **Correspondence:** Cynthia Formosa

**Background:** Depression and diabetes have ranked amongst the defining epidemics of the 21st century, given the current explosion in the prevalence rates of both these conditions, therefore it is not surprising that these two conditions could interact when both are present, leading to additional morbidity and a higher mortality risk in patients living with diabetes. Despite this, the specific relationship between depression and DPN remains unclear. This study aimed to determine any relationship between having diabetic peripheral neuropathy and the development of depressive symptoms in patient with type 2 diabetes mellitus (T2DM).

**Methods:** A comparative non-experimental study was conducted. Ninety five T2DM individuals aged 65 years and more were recruited. The sample was divided into two groups; 50 participants with T2DM only and 45 participants with DPN. The Patient Health Questionnaire-9 (PHQ-9) was used to collect information about low mood/depression symptoms in the recruited subjects.

**Results:** Participants with DPN recorded higher scores of PHQ-9 than those with T2DM only. The mean PHQ 9 score for the Diabetic Peripheral Neuropathy group (6.09) was significantly higher than the mean PHQ 9 score for the T2DM group (2.24) (p<0.001). Participants with DPN were more likely to have a mild/moderate or moderately severe low mood/depression symptoms, when compared to Type 2 DM participants who exhibited minimal to no low mood/depressive symptoms.

**Conclusions:** The association between diabetic neuropathy and depression is confirmed, with significant depressive symptoms found in patients with neuropathy when compared to patients with diabetes with no complications. Therefore, complaints caused by DPN and emotional problems associated with DPN should be addressed in the management of DPN in order to prevent depressive symptoms. A call for change in screening practices to help identify patients with DM and depressive symptoms is warranted.

### C7

#### C7.5 Using a modified nominal group technique to develop complex interventions for a randomised controlled trial in children with symptomatic pes planus

##### Mike Backhouse^1^; Daniel Parker^2^; Stewart Morrison^3^; Jennifer Anderson^2^; Sarah Cockayne^4^; Joy Adamson^4^

###### ^1^University of Warwick, Warwick, UK; ^2^University of Salford, Salford, UK; ^3^Brighton University, Brighton, UK; ^4^University of York, York, UK

####### **Correspondence:** Mike Backhouse

**Background**: Children with symptomatic pes planus frequently present for care but there remains uncertainty about how best to manage them. Currently, management varies considerably within and between professions. Corrective surgery remains rare but exercise, foot orthoses, and advice regarding suitable footwear are commonly provided in line with the international consensus. We intend to test frequently used interventions (exercise and advice, exercise and advice plus prefabricated insoles, and exercise and advice plus custom made insoles) in a subsequent RCT. Each of these interventions are multifaceted and considered complex so require developing prior to starting the trial.

**Methods:** We used a modified Nominal Group Technique combining an electronic survey with two face-to-face meetings to achieve consensus on the final logic model and menu of options for each intervention.

**Results**: In total 16 healthcare professionals took part in the consensus meetings (seven at Eastbourne, and nine in Salford). These consisted of 11 podiatrists, two orthotists, two physiotherapists, and one orthopaedic surgeon.

Both meetings endorsed the logic model with amendments to reflect the wider psychosocial impact of pes planus and its treatment, as well as the increasing use of shared decision making in practice. Short lists of options were agreed for prefabricated and custom-made insoles, structures to targe in stretching and strengthening exercises, and elements of health education and advice.

**Conclusions**: Our novel modification of the nominal group technique produced a coherent logic model and shortlist of options for each of the interventions that explicitly enable adaptability. We formed consensus on the range of what is permissible within each intervention so that their integrity is kept intact, and they can be adapted and pragmatically applied. The process of combining survey data with face-to-face meetings has ensured the interventions mirror contemporary practice and may provide a template for other trials.

#### C7.6 Foot development: a narrative synthesis of plantar pressure patterns during infancy and childhood

##### Eleonora Montagnani^1^; Carina Price^2^; Christopher Nester^2^; Stewart Morrison^1^

###### ^1^School of Health Sciences University of Brighton, Brighton, UK; ^2^School of Health and Society University of Salford, Salford, UK

####### **Correspondence:** Eleonora Montagnani

**Background:** Quantifying plantar pressure throughout periods of development enables us to gain an understanding of typical changes in foot function occurring alongside motor development. The aim of this review was to synthesise existing study outcomes, which report plantar pressure patterns of infants and children, to explore changes in foot function occurring throughout childhood.

**Methods:** A narrative approach was adopted, and a literature search undertaken using three research platforms: Science Direct, PubMed, Google Scholar. From the search, 263 articles were found. Articles were included if written in English, included experimental plantar pressure pattern data, and recorded data with electronic pressure measurement systems, from typically developing feet of infants and children up to 13 years of age. Fifteen articles met the inclusion criteria and were included in the review.

**Results:** By scoping the literature, plantar pressure values (e.g. contact area, maximum force) were found to increase in most foot regions with age but decrease at the midfoot during infancy and childhood. Thus, plantar pressure patterns evolve as infants and children become more proficient in walking, highlighting a trajectory of foot development in infants and children. Several limitations of existing studies were also identified including the use of cross-sectional designs and inconsistencies in data collection protocols and data analysis. No reports were identified of earliest stages of foot function evolution.

**Conclusions:** Further work could describe how the foot develops and experiences load prior to walking and as independent walking evolves, providing the scientific community with baseline knowledge of foot development.

#### C7.7 Gait development in infants: reflections on real-world data collection and analysis in the Small Steps study

##### Carina Price^1^; Eleonora Montagnani^2^; Chris Nester^1^; Stewart Morrison^2^

###### ^1^School of Health and Society, University of Salford, Salford, UK; ^2^School of Health Sciences, University of Brighton, Brighton, UK

####### **Correspondence:** Carina Price

**Background**: Robust knowledge of foot development during infancy is lacking and coupled with recent lessons from developmental psychology literature, alternative approaches to biomechanical evaluation of gait in infants are needed. Small Steps was a five-year funded research programme, (launched 2016) to investigate changes occurring in infant feet as they develop as weight-bearing structures. The purpose of this abstract is to share our reflection on data collection with infants.

**Methods**: A two-site longitudinal study was designed with four visits representing specific motor development milestones (reach for feet, pull-up to stand, onset of walking and confident walking). Across both sites, we designed modified gait labs (baby spaces) to capture real-world motion strategies, which have high external validity and represent typical bouts of infant walking. Our data collection space included a gated area, creche flooring and central pressure platform surrounded by motion capture cameras. Tasks were performed on the pressure platform at each visit and during the final two visits (once the infant was walking) kinematic data were also collected. Each visit also included measurement of anthropometrics and skin properties.

**Results**: 131 infants attend the first visit of the longitudinal study, within 21 days of reaching for their feet. 78 of these infants complete the fourth visit, within 21 days of being stable confident independent walkers. Participant loss was due to drop-out, exclusion and ceasing testing due to the COVID-19 pandemic.

**Conclusions**: The longitudinal study design was challenging in terms of recruitment and continued engagement with families. Booking visits close to milestone achievement was also difficult to schedule. Activities during data collection required effective explanation to parents and being sensitive to concerns alongside age-appropriate skills to keep infants engaged. Novel approaches to data analysis have been implemented such as floating lab coordinate systems for kinematics and personalised age-appropriate masks for pressure analysis.

#### C7.8 The prevalence and impact of ankle haemarthrosis in moderate and severe haemophilia A and B; The HAPII study

##### Richard A Wilkins^1^; Heidi J Siddle^2^; Graham J Chapman^3^; Elizabeth Horn^4^; Ben Palmer^5^; David Stepehensen^6^; Anthony C Redmond^2^

###### ^1^Leeds Institute of Rheumatic and Musculoskeletal Medicine, University of Leeds, Leeds, UK; ^2^Leeds Institute of Rheumatic and Musculoskeletal Medicine, University of Leeds, Leeds UK; ^3^School of Sport and Health Sciences, University of Central Lancashire, Preston, UK; ^4^Leeds Haemophilia Comprehensive Care Centre, Leeds Teaching Hospitals NHS Trust, Leeds, UK; ^5^UKHCDO, National Haemophilia database, Manchester, UK; ^6^Kent Haemophilia and Thrombosis Centre, East Kent Hospitals University NHS Trust, Canterbury, UK

####### **Correspondence:** Richard A Wilkins

**Background:** Haemarthrosis is an inherent clinical feature of haemophilia A and B [1]. Whilst annual joint bleed rates are commonly reported, there is a lack of information on bleed rates, joint health in individual joints and the impact of ankle haemarthropathy [2].

**Methods**: Ethical approval was obtained (IRAS: 206141, R&D:PD16/227) A nationwide prevalence study was undertaken in conjunction with the United Kingdom Haemophilia Centres Doctors Organisation, National Haemophilia Database. A further multicentre study to determine the impact of ankle haemarthropathy was undertaken across 18 haemophilia centres in England, Wales and Scotland.

**Results:** Prevalence: 2238 cases were identified; 273 reported ≥75% simultaneous Haemtrack compliance and Haemophilia Joint Health Scores (HJHS) data. Higher HJHS were reported for the ankle joint compared to the knee and elbow, confirming that the ankle joint is the most affected joint in people with haemophilia.

Impact: 244/273 participants with ankle haemarthrosis reported poor quality of life (QoL) regardless of haemophilia type (A,B), severity (moderate,severe) or treatment (on demand,prophylaxis). MOXFQ scores were poor, similar to scores seen in patients with ankle osteoarthritis listed for fusion surgery. Most participants reported no access to podiatry (58%) and no use of foot orthoses (51%), or modified footwear (88%).

**Conclusions**: In adults the ankle is the most common site of haemarthrosis and is disproportionally affected by haemarthropathy. The associated impact is severe, and the new data provides a benchmark to potentially evaluate emerging pharmacological and targeted non-pharmacological treatments. Interventions such as foot orthoses and modified footwear have a role in delaying haemarthropathy and improving QoL.

#### C7.9 Patients' and clinicians' perspectives on the clinical utility of the Rheumatoid Arthritis Foot Disease Activity Index

##### Anika Hoque^1^; Gordon Hendry^1^; Diane Dickson^2^; Martijn Steultjens^1^

###### ^1^School of Health and Life Sciences, Glasgow Caledonian University; ^2^School of Health and Life Sciences – Diagnostic Imaging, Glasgow Caledonian University- Diagnostic Imaging.

####### **Correspondence:** Anika Hoque

**Background:** The Rheumatoid Arthritis Foot Disease Activity Index (RADAI-F5) is a valid PROM for measuring foot disease activity in rheumatoid arthritis (RA). This study aims to understand the clinical potential of the RADAI-F5 as a tool to inform management.

**Methods:** 60-minute, semi-structured, one-on-one Microsoft Teams interviews with adult RA patients and rheumatologists, physiotherapists, and podiatrists involved in treating RA patients was conducted as part of qualitative research. Interviews involved open-ended questions to explore barriers and facilitators to the clinical application of the RADAI-F5 and were audio-recorded and transcribed verbatim, with each participant verifying the transcript to establish rigour. Inductive thematic data analysis was applied using Nvivo11.

**Results:** Eight RA participants: 7 females; mean [standard deviation, SD] age 52.4 [9.5], mean [SD] disease duration 16.1 [16.4] and eight clinicians; mean [SD] age 46.75 [5.3], mean [SD] years of clinical experience of 19.5 [2.21] participated. Four main themes supporting the clinical utility of the RADAI-F5 were identified. These included; 'clinical feasibility' as the tool is short and quick to complete; 'promoting patient-clinician communication' as it can identify areas of concern that may be under-recognised by clinicians; 'monitoring status and treatments longitudinally' as implementing the RADAI-F5 could inform an individuals' management and; 'patient involvement,' since the tool may trigger patients to be more involved in their foot health self-management. Three themes were identified as potential barriers to implementation; 'practical difficulties, including lack of time during appointments; 'lack of validity', as clinicians were hesitant to use the RADAI-F5 until its validity is established further; and, 'lack of an electronic database', since there is currently no electronic system for reporting RADAI-F5 results.

**Conclusions:** The RADAI-F5 has significant potential as a clinical tool to aid foot disease management in RA. Nevertheless, some barriers to implementation need to be addressed to enable widespread use in rheumatology clinics.

### POSTER PRESENTATIONS

#### P.10 Foot emergency action team (feat) - managing diabetes foot complications in the clinically vulnerable during covid-19

##### Jessica Bolton; Paul Taylor; Paul Dee; Joelle Baynham

###### Dorset Healthcare University NHS Trust, Dorset, UK

####### **Correspondence:** Jessica Bolton

**Background:** As the country went into lockdown on 23rd March 2020, and people that were identified as being clinically at risk of Covid-19 were advised to stay at home, we were faced with the dilemma of how we could provide care for our most complex diabetes foot clinic patients to prevent foot related hospital admissions and amputations? The Foot Emergency Action Team (FEAT) was set up. It was staffed by Specialist Diabetes Podiatrists from the Diabetes foot team who visited these patients in their homes with remote support from Consultant Diabetologists, vascular surgeons, and Advanced Podiatrists. Robust treatment plans were put in place to manage these patients without attending the diabetes foot clinic. The aims where to:

- Prevent hospital admissions due to foot complications;
- Prevent avoidable amputations;
- Prevent avoidable deaths from diabetes foot complications;
- Enable these complex patients to shield from Covid-19 as per government guidelines.

**Methods:** The FEAT home visits ran from April 2020-November 2020. During this time we had a total case load of 34 patients.

**Results:** At the start 85% (N=29) had severe ulceration (SINBAD score 3-6), which reduced to 50% (N=17), an improvement of 35%. During this time 27% (N=9) healed, and 29% of patients (N=10) had an improved SINBAD score. There were 6 deaths (18%), none foot related, and one was due to Covid-19.Two admissions occurred (6%), one was for a BKA due to osteomyelitis of the fibula. The other admission was due to sepsis that was picked up on the patients first FEAT visit.

**Conclusions:** The FEAT visits were an effective way of providing a high level of holistic diabetes foot care in the patient’s home, helping to prevent hospital admissions and amputations, whilst also protecting them from the risk of covid-19 infection.

#### P.11 Pilot patient study investigating the impact on the provision of care of active diabetic foot disease during peak COVID-19 pandemic restrictions

##### Sinead Flynn; Caroline McIntosh

###### Discipline of Podiatric Medicine, National University of Ireland, Galway, Ireland

####### **Correspondence:** Sinead Flynn

**Background:** The extensive impact COVID-19 has had on the provision of care in outpatient departments has been an especially concerning time for service users. This has been a particular worrying time for those living with active diabetic foot ulceration who rely on regular consultations with their foot protection team. The aim of this pilot study was to investigate the patients perception on the changes to the provision of care during the government imposed pandemic restrictions and establish the impact this had on the physical and psychosocial wellbeing of this patient cohort.

**Method:** Patients who had active diabetic foot disease prior to the first pandemic lockdown on the 16th of March 2020 or who developed a diabetic foot ulcer during the pandemic were invited to participate at their routine appointment. This study consisted of a questionnaire with participants also given the option to partake in a semi structured interview to further elaborate on their experiences during the COVID-19 pandemic.

**Results:** The patient questionnaire and the semi structured interview yielded 30 and 5 responses respectively. No sophisticated data analysis has been completed, however preliminary findings have shown that patients are highly satisfied with the provision of care despite the necessary changes imposed by the COVID-19 restrictions. Contrary to this, clinical outcomes as reported by the patients do not reflect such a positive trend, with many patients living with their diabetic foot ulcerations much longer than the norm as stated by the literature. Loneliness and social isolation were the most common

**Conclusions:** The relocation of more services out to the community were positively welcomed by service users however the patient self-reported outcomes do not correspond. This highlights the need for improved community-based care which would also help disburden the overcrowded hospital clinics.

#### P.12 Routine fluorescence imaging to detect wound bacteria reduces antibiotic use and antimicrobial dressing expenditure while improving healing rates: Retrospective analysis of 229 foot ulcers

##### Nadine Price

###### North East London NHS Foundation Trust, London, UK

**Background**: Foot ulcers and their bacterial burden produce a significant strain on the NHS. Subjectivity of wound infection assessment makes appropriate dressing selection challenging. This retrospective pre/post-analysis evaluated how implementation of fluorescence imaging impacted (1) antimicrobial dressings and antibiotics use and (2) wound healing rates.

**Methods**: To aid point-of-care detection of bacterial burden, a fluorescence imaging device (MolecuLight i:X) was introduced to our high risk foot clinic. Over a 2-year period 229 lower extremity wounds were treated. Wound-related outcomes and antimicrobial dressing costs were quantified over 1-year before (2018/2019) and after (2019/2020) incorporating fluorescence imaging into routine practice.

**Results**: The period of fluorescence imaging saw a 27% increase in the number of wounds seen, yet annual antimicrobial dressing expenditure decreased by 33%. Implementation of fluorescence imaging was also associated with a 49% decrease in prescription of antimicrobial dressings, a 33% decrease in antibiotic prescriptions, and a 23% increase in wound healing rates within 12-weeks (48% vs. 39%), likely due to earlier bacterial detection and improved wound hygiene. This increased healing rate is projected to decrease annual wound costs by 10% (£762 per patient).

**Conclusions**: Routine bacterial imaging appears to diminish clinical and economic burden to patients and the NHS.

#### P.13 Reducing MINOR Amputation Rates in A Diabetes Limb Salvage Service (DLSS) Root Cause Analysis (RCA) to Guide Service Improvement

##### Thomas Dickie^1^; David Russell^2^; Heidi Siddle^1^

###### ^1^Leeds Teaching Hospitals NHS Trust, Leeds, UK; ^2^ School of Medicine, University of Leeds, Leeds, UK

####### **Correspondence:** Thomas Dickie

**Background:** Over 7,000 diabetes-related amputations occur yearly having enormous impacts (short and long term) on quality of life for patients, family/carers. Diabetic foot minor amputation (DFMinA) rates in Leeds (23.4 procedures per 10,000) is higher than the national average (21.4 per 10,000)^3^; 108 reported minor amputations were performed 2018-2019, a 52% rise from 2017-18. The aim of the project was to undertake an RCA exploring contributing factors for DFMinA in Leeds. The objectives were to (1) Identify RCA tool

The Yorkshire and Humber’s Diabetic Foot Network RCA tool (for Major amputations) was utilised and (2) to determine the appropriateness of RCA tool.

**Methods**:

- Retrospective Quantitative and Qualitative Design
- Patients identified via Hospital coding April 2018 - Oct 2018
- Electronic Hospital and Primary care records were reviewed

**Results:** An overview of the results are provided in table 1, figures 1 and 2.

**Conclusions:** A large proportion of DFMinA appear to be caused by a lack of access/early treatment or no preventative treatments (71%). Findings are consistent with outcomes reported (EURODIALStudy^1^), indicating that DFMinAs could be prevented with early presentation to foot care services. This RCA indicates that prioritising early presentation, targeting deprived areas, fast tracking PAD assessment/intervention and providing correct treatment for infection could reduce diabetic foot minor amputation rates in Leeds. This RCA tool appears an appropriate tool assisting DFU services to understand DFMinA.

**Table 1 (abstract P.13).**
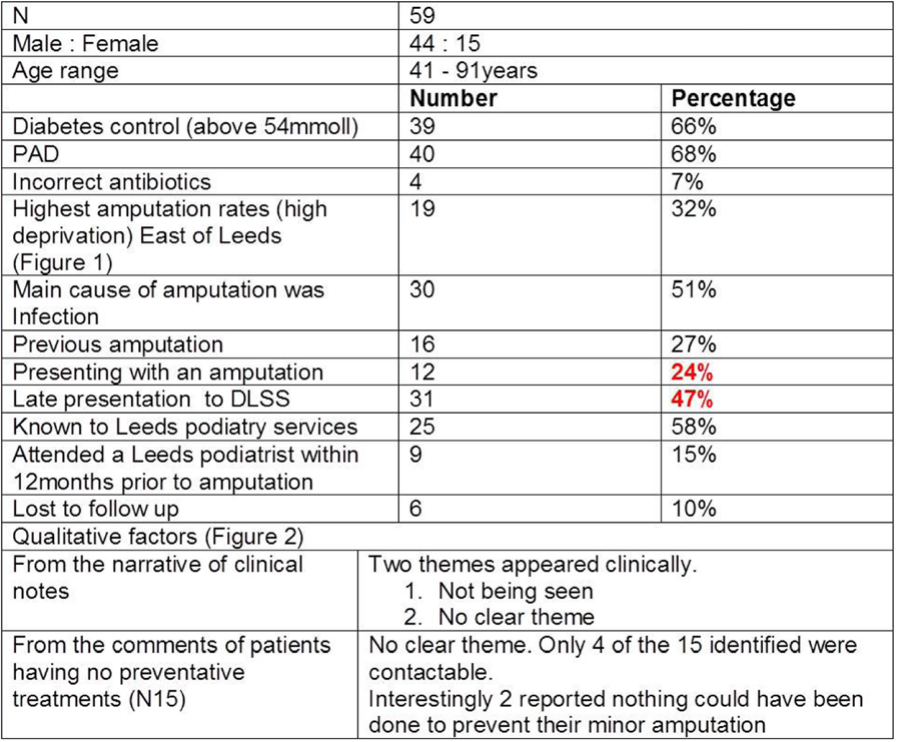
Patient demographics, presentation and services

**Fig. 1 (abstract P.13).**
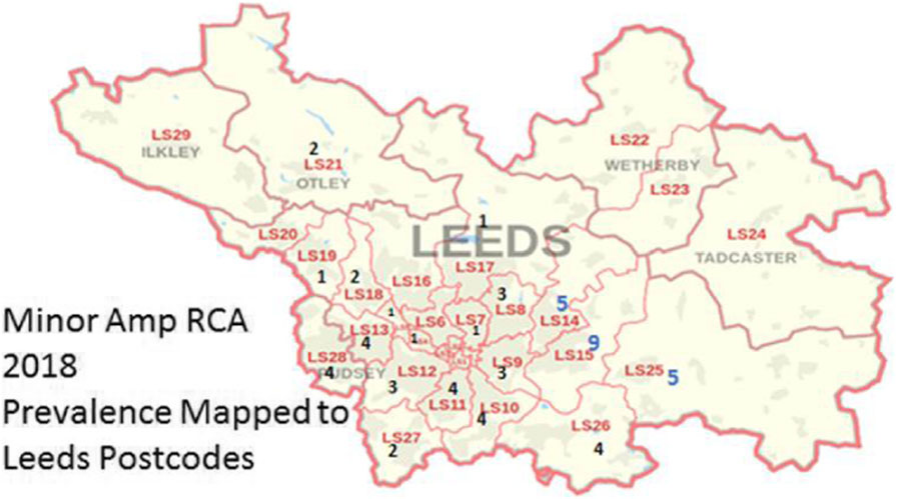
Prevalence of minor amputations (Leeds Postcodes)

**Fig. 2 (abstract P.13).**
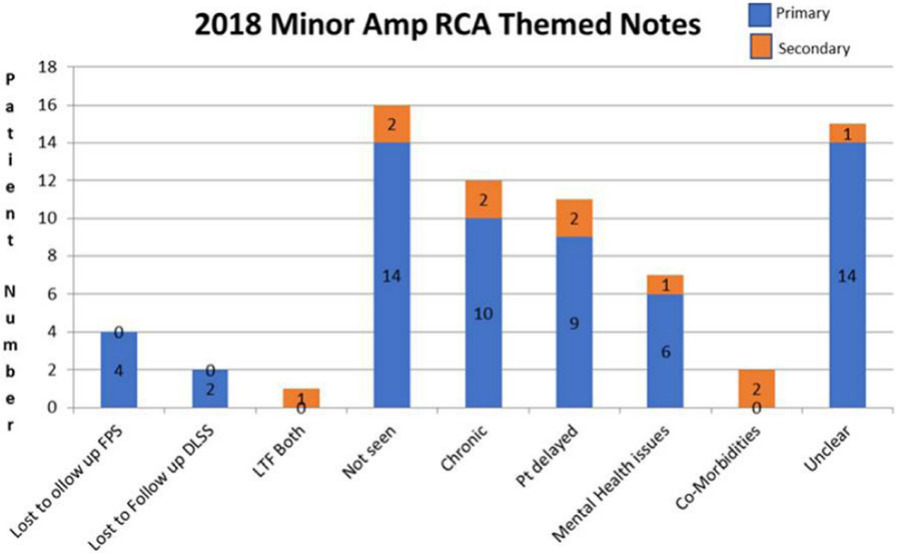
Minor amputation (2018) themed notes

#### P.14 Foot morbidity in patients with ESRD on dialysis

##### Nathalie Schembri

###### Cynthia Formosa, Faculty of Health Sciences, University of Malta, Msida, Malta

**Background**: To determine the prevalence of foot morbidity amongst patients with ERSD on dialysis treatment.

**Method**: Forty seven patients were recruited from the Renal Unit, at the National Hospital, Malta. A non-experimental, non-randomized quantitative time series design was employed. Information was gathered via an initial consultation followed by various non-invasive foot assessments: Neurological, Arterial, Biomechanical, and Dermatological Assessments.

**Results**: The findings demonstrated high prevalence of foot morbidity amongst this population. 95.74% presented with foot deformities, whilst 76.60% presented with skin and nail conditions. 22% had a history of ulceration, 19% had a history of amputation, 9% had active ulceration, and 7% had history of revascularisation. Findings demonstrated poor foot-care behaviour with 40.43% presenting with inappropriate footwear, 70% did not check feet regularly, 87% did not attend to podiatry appointments, whilst 68% were unable to reach their feet for self-care. The mean TBPI decreased with time in this cohort. The relationship between the TBPI and duration of dialysis was found to be statistically significant. Both DM and dialysis duration were identified as significant predictors for the reduction in TBPI. Results indicated that for every one month increase in dialysis duration, the TBPI was expected to decrease by 0.013 and that the mean TBPI for patients with DM and ESRD was expected to be 0.1565 less than the mean TBPI of patients with ESRD.

**Conclusions**: This study highlighted the importance of expanding practice by introducing a new podiatry service within the renal unit to provide prompt foot screening, foot care, and foot care education, with the aim to reduce severe foot complications. This study recommended that ESRD patients on dialysis should embark on a podiatry screening algorithm as soon as they are diagnosed with this condition and continue to be monitored closely within the renal unit to delay and/or prevent severe outcomes.

#### P.15 How reliable is your National Diabetic Footcare Audit (NDFA) data?

##### Thomas Dickie^1^; Begonya Alcacer-Pitarch^2^; Helena Meally^2^

###### ^1^Leeds Teaching Hospitals NHS Trust, Leeds, UK; ^2^Leeds Institute of Molecular Medicine, University of Leeds, Leeds, UK

####### **Correspondence:** Thomas Dickie

**Background:** The National Diabetic Foot Audit data (NDFA) is a national database that collects continuous data for specific clinical outcomes of diabetic foot management. The outcomes have three key areas: *structures* (1), *processes* (2) and *outcomes* (3).

**Methods:** A recognised limitation from NDFA data is its reliability. The NDFA authors acknowledge the ascertainment figures influences the outcomes. *"Probable low case ascertainment should therefore be considered when interpreting NDFA findings"* There is large variation on the number of DFU episodes entered from different service providers. From 2018 to 2019 entry results varied from 640 episodes to zero. An element of selection bias should be considered when interpreting the data, especially when isolating one’s own service data comparing to others. The aim of this project was to identify which NDFA outcomes were incomplete for the LTHT DLSS service.

**Results:** Figure 1 represents episodes following cross referencing LTHT electronic health records with NDFA data. This was then repeated following service changes to facilitate improvements. This factored a 94% improvement.

**Conclusions:** The audit supports improvements were necessary to increase ascertainment and reliability.

Possible strategies to help improve NDFA inputting:-

1. Use tools to measure i.e. year end audit
2. Encourage evidence based outcomes from reliable data
3. Review the national picture

**Fig. 1 (abstract P.15).**
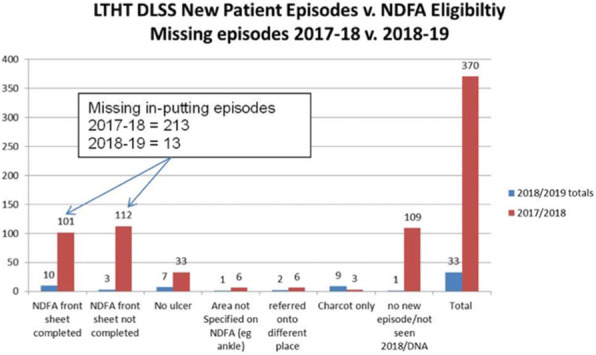
Cross referenced NDFA and DLSS missing episodes

#### P.16 Shared patient and multidisciplinary foot team experiences in using a hands-free single crutch as a novel therapy in the post-operative rehabilitation of diabetes related foot complaints

##### Daina Walton^1^; Joanne Casey^1^; Chris Manu^1^; Prashanth Vas^1^; Christian Pankhurst^2^; Charlotte Hobbs^1^

###### ^1^Diabetes Foot Clinic, King's College Hospital NHS Foundation Trust, London, UK; ^2^Community Rehabilitation Services, Department of Orthotics, Guy's and St. Thomas' NHS Foundation Trust, London, UK

####### **Correspondence:** Joanne Casey

**Background:** To report patient and health care practitioner (HCP) experiences in the use of a hands-free single crutch (HFSC) as a novel therapy in the post-operative rehabilitation of diabetes related foot complaints. This is a removable device that can be attached to the thigh and transmits weight through a flexed knee. This enables the individual to remain independently mobile and not bear weight through the injured foot.

**Methods:** Over 18-months, six inpatients chose to self-purchase an HFSC for use in their post-operative rehabilitation. These patients selected this device as it maintained their independence in mobility and was an alternative to the housebound, non-weight-bearing, microenvironment advised for their recovery. During inpatient therapy, an ability to mobilise along a 100-metre walkway with the HFSC was undertaken before discharge to ensure patient safety in mobilisation.

**Results:** Within this cohort, 83% were male, 67% had Type 2 Diabetes, age (mean±SD) 55±11 years and HbA1c (mean±SD) 81±26 mmol/mol. Pre-admission, all patients were fully weight-bearing independently except for one who was performing standing transfers only. The reason for admission were three elective reconstructions, two emergency minor lower extremity amputations and one for calcaneal osteomyelitis.

For the 100-metre walkway test, four patients were independent in using the HFSC, one patient used the HFSC and two elbow crutches, and one patient was unable to use the HFSC with or without support. No falls were observed during their admissions. Five patients were discharged using the device. On outpatient follow up at 12 weeks, two continued to mobilise independently with the device.

**Conclusions:** This observational case series using the HFSC as a novel therapy requested by patients in the post-operative rehabilitation of diabetes-related foot complaints found mixed results. HCPs should be aware of its strengths and limitations.

#### P.17 DRIVES STUDY: Differences in MDT Referrals in Infections and Vascular Evaluation Study. Evaluation of the extent COVID-19 has impacted referrals to diabetic Multi-Disciplinary Team (MDT) services from Podiatric services and clinical outcomes of patients within an English community NHS Trust

##### Ella Reid; Warren Song; Lindsey Cherry; Lucy Gates

###### School of Health Sciences, University of Southampton, Southampton, UK

####### **Correspondence:** Ella Reid

**Background:** COVID-19 lockdown resulted in reduced NHS Podiatric service provision in several treatment centres. We hypothesised that in a single centre, despite high-risk services remaining available, a reduction in multidisciplinary consultation or clinical outcome for people at higher risk of amputation could result because of wider service restriction. This service evaluation aimed to quantitatively investigate multi-disciplinary team referral differences between March-July 2019 and March-July 2020 and qualitatively explore root causes of below knee amputation in a single 2020 case-based example.

**Methods:** A two-stage, mixed methods, evaluation of 1) Diabetic foot MDT referral within a single South-England NHS centre was carried out between 2019 and 2020, and 2) a case-based root cause analysis. Outcome variables included SINBAD score (0-6), clinician-defined ulcer aetiology (0-3), vascular status (0-2), neurological status (0-1) and reason for referral. Tests of statistical difference or association were completed using Mann-Whitney U-test or Pearson’s Chi-square analysis. An RCA case exemplar was randomly selected from the 2020 cohort. Data triangulation was undertaken to determine potentially modifiable factors to case or cohort outcomes.

**Results:** In 2019 and 2020, 414 and 284 referrals were made respectively (40% reduction). There was a significant decrease in the percentage of patients whose primary reason was recorded as “other” in 2020 compared to 2019 (p=0.008). The mean SINBAD score was significantly higher for referrals in 2020 than 2019 (p=0.019). Reduced shared decision-making in care during COVID-19 restrictions was identified as possibly contributing to poorer clinical outcomes.

**Conclusions**: COVID-19 allowed us to investigate how disruption to breadth of podiatric service provision can impact patient outcomes. Our evaluation has quantified a reduction in high-risk case MDT referral, a worsening in foot health at the point of referral, and uncovered a negative impact upon shared decision-making. This highlights important considerations for Podiatric service design post–COVID-19.

#### P.18 Trigger hallux in the diabetic patient: surgical options and case presentation

##### Matthew Mee; Ewan Kannegieter

###### Provide CIC Podiatric Surgery, Braintree, Essex, UK

####### **Correspondence:** Matthew Mee

**Background:** A poster presentation outlining anatomy, definition, aetiology, pathophysiology and surgical management options.

**Methods:** Case presentation with clinical pictures and X-Rays; pre op, post op, and at 6 weeks.

**Results:** The case highlights a management option to a common clinical problem encountered in patients who have diabetes that can cause recurrent ulceration and resultant morbidity.

**Conclusions:** The case was referred from our community podiatry team and serves as an example of how podiatrists and podiatric surgery teams can work synergistically to offer solutions to potentially limb threatening conditions.

#### P.19 The relationship between plantar foot lesion patterns and first metatarsophalangeal joint (MTPJ) mobility

##### Jennifer Topalian

###### Cardiff Metropolitan University, Cardiff, UK

**Background:** Plantar lesions are an important clinical symptom of 1st MTPJ pathology, indicating high weight-bearing pressure areas and possible compensatory foot function in gait. Research has yet to link the severity of *hallux limitus* (HL) and plantar foot lesion patterns. Where plantar lesions have been mentioned, they are not related to HL severity. To investigate the relationship between plantar foot lesion patterns and severity of weightbearing 1st MTPJ dorsiflexion restriction. To identify potential pressure areas that may need to be offloaded to prevent ulceration and help validate lesion patterns as a clinical marker of HL severity.

**Methods:** A cross-sectional, correlational study using a convenience sample of 35 participants, using the ball kick test to determine foot dominance, the Jack’s test plus DrGoniometer application to measure weightbearing 1st MTPJ dorsiflexion and photographing plantar foot lesions.

**Results:** No clear link was found between amount of weightbearing 1st MTPJ dorsiflexion and the number or location of plantar foot lesions. Five pattern trends emerged, but the frequency of these was low and the standard deviation from the mean dorsiflexion was high, except for of the pattern of: 1st IPJ, 2nd, 3rd, and 5th MTPJs.

**Conclusions:** The results of this study agree with the literature with fewer lesions found under the 1st MTPJ than the lesser met heads and 1st IPJ. A possible significant pattern trend was 1st IPJ, 2nd, 3rd, and 5th MTPJ, as mean dorsiflexion standard deviation was small. Further research using a larger, and broader sample is needed to investigate this.

#### P.20 Neurospecific Foot Mobilisation: towards a biological plausibility hypothesis of a passive

##### intervention in relation to adult post-trauma dystonic foot and ankle presentations

###### Ian Linane

####### PodiaClinic Ltd, Brighton, UK

**Background:** Dystonia of the foot and ankle is a life altering condition and a rare presentation in podiatry clinics.

**Methods:** Between 2014-21 four such conditions presented in a private podiatry context, were successfully treated, the outcomes of which fed into 7 years of reflective practice, in turn driving and guiding development of a novel passive stimulus approach to their treatment: Neurospecific Foot Mobilisation (NSM).

**Results:** NSM is primarily a tissue mobilisation intending to target Ruffini afferents, classed as Slow Adapting type 2 (SA2), within the cutaneous and fascial tissues of the plantar medial foot arch and ankle retinacula. To move this approach into possible pilot studies there is need to establish, at minimum, a neurobiological plausibility to underpin it. That is, there is presence of relevant afferents with neuromodulating capacity in the mobilised tissues. Drawing on cutaneous and muscle microneurography, neuroanatomy of fascia, current concepts of Manual Therapy (MT) and case reflection, this poster explores such a possibility.

**Conclusions:** NSM’s gentle application serves as a standalone treatment or as part of a multimodal care pathway. It is proposed as a specific BIO within the Biopsychosocial framework. To date, a search of the literature has not brought up a similar reported approach nor associated outcomes.

#### P.21 Comparing the amount of contractility in elastic 'kinesiology' tape when applied to the thinner skin on the lateral surface of the leg and the thicker skin on the plantar surface of the foot at maximum stretch

##### Alec Pendrill; John Tasker

###### Birmingham School of Podiatry, Birmingham Metropolitan College, Birmingham, UK

####### **Correspondence:** Alec Pendrill

**Background:** Elastic ‘kinesiology’ tape is commonly used in the management of acute and chronic musculoskeletal disorders. The therapeutic action of elastic tape occurs when tape is applied to the skin under different percentages of stretch, contracting on the skin, creating wrinkles in the skin layers and fascia and theoretically decompressing deeper structures e.g. muscle tissue. As plantar foot epidermis is thicker than non-weight bearing epidermis e.g. on the lateral side of the leg then, is the contractility of the tape affected when applied to different thicknesses of skin

**Method:** Using a within subject, quasi-experimental study with convenience sampling, n=14, 10 healthy females and 4 healthy males, age range 18-61 years, each subject was exposed to a control test, where the tape applied to the 2 types of skin without stretch and two test conditions:

1. tape is applied at full stretch on plantar skin
2. tape is applied at full stretch on the non-weight bearing skin.

**Results:** Using a one-tailed paired t – test, significance level p<0.05, it was inferred that there is a highly statistically significant difference in contractility of the tape, the amount of contraction of elastic tape was greater on the thinner lateral skin than on the thicker plantar skin (mean difference = 0.35cm, t = -7.92, 95% Confidence Interval: 0.26 to 0.45cm, p<0.00001)

**Conclusions:** Elastic tape exhibits a greater contraction from full stretch when applied to thinner skin compared to thicker skin.

#### P.22 Foot skin hydration: Quantification, interpretation and opportunities for modification

##### Jennifer Andrews

###### EPSRC Centre for Doctoral Training in Prosthetics and Orthotics, University of Salford, Salford, UK

**Background:** The human foot is a common location for anhidrotic skin pathology, despite this there is a paucity of data available on foot skin hydration. The limited data that are available are also of unknown value as these are obtained from a consistently shallow depth within the stratum corneum, a structure known to vary in thickness across the foot, the hydration gradient of which is unexplored in this location. To identify aberrant skin hydration and evaluate treatment efficacy, understanding physiological hydration of foot skin is essential. Due to the unique and varied anatomy of foot skin, examining the stratum corneum hydration gradient across the foot, and how this is represented by commonly used hydration measurement techniques, is required to resolve uncertainty currently associated with foot skin hydration measurements.

**Methods:** As part of a PhD programme funded by Scholl’s Wellness Co, two studies will be undertaken:

**A**: In-vivo Raman Spectroscopy will be used to examine the depth profiles of water and lipids within the epidermis of 6-8 participants at multiple locations across the foot (plantar and dorsal).

**B**: 80 participants aged 20-40 years of age will have hydration of their skin measured at several locations (foot and body) using three devices that utilise different mechanisms for quantifying tissue water.

**Results:** Measures of other tissue characteristics known to correlate with water content will be collected simultaneously.

A: Novel insight into the composition of the foot skin.

B: Foot-specific knowledge of the link between skin hydration and physical characteristics.

A+B: Understanding of how skin composition influences skin characteristics and how this is represented by hydration measurement devices.

**Conclusions:** These data will inform how foot skin hydration is quantified and interpreted in future using instruments that are suitable for use within a clinical and commercial environment. This will support the evaluation of treatment efficacy for anhidrotic foot pathologies.

#### P.23 Developing a career long mentorship framework

##### Emma Noe; Robin Hull

###### Podiatry Department, Harrogate and District NHS Foundation Trust, Harrogate, UK

####### **Correspondence:** Robin Hull

**Background:** It has been identified through staff survey and focus groups that although HDFT Podiatry Services provides a widely acknowledged induction and preceptorship package for new graduates there is a lack of structure in mentorship and career development strategies for established clinicians. Little training is dedicated in preparing clinicians to become mentors themselves.

**Methods:** A development day was help with Band 7s across the service. A standardised mentorship framework was developed to ensure all staff, regardless of Band or aspiration have access to the same support. Standard operating procedures and mentorship contracts were devised.

**Results:** The framework was rolled out service wide in October 2019. Regular updates and training days are to be held to support those delivering the support. Initial feedback indicates a positive effect of recruitment, retention and morale. Clinicians feel well supported and Band 7s feel empowered within their leadership roles.

**Conclusions:** Ongoing work is to be done to continue to evaluate and develop the program

#### P.24 Motivations for career choice for podiatry students in England: Findings from a national questionnaire

##### Lucy Wallis^1^; Rachel Locke^1^; James Faulkner^1^; Beverley Harden^2^

###### ^1^Faculty of Health and Wellbeing, University of Winchester; ^2^Health Education England, London, UK

####### **Correspondence:** Lucy Wallis

**Background:** Addressing shortages for podiatrists is a goal of the NHS Long Term plan. However, in recent years there has been reductions in the numbers choosing to study the subject in the UK. The aim of this research was to understand students’ motivations for choosing a podiatry career, their sources of influence and barriers to entry. Understanding career decision-making processes will inform healthcare career promotion and advice for podiatry courses.

**Methods:** An online questionnaire was disseminated to podiatry students in England between February and March 2021. This comprised demographic and Likert scale questions, with open-ended questions about public perception of podiatry and advice to individuals interested in the profession. Descriptive analysis was applied to the data.

**Results:** The questionnaire was completed by 115 podiatry students. Female students comprised 87% of the respondents and 75% of respondents were white. Of the sample, 38% were under 25. Choosing podiatry to improve the quality of life for a patient/service user was the most influential motivation. The biggest source of influence on selecting a podiatry career was from conducting their own research. Misconceptions around the profession and what the role involves was seen as the most significant barrier to choosing the career.

**Conclusions:** As has been highlighted in other studies, a lack of awareness and information about podiatry were identified as the key barriers to choosing podiatry. This was evident in personal sources of influence, such as meeting a podiatrist, as the most important sources of influence. On the whole, educational, media and marketing sources scored low in terms of influence. The findings highlight areas of investigation for future research. For example, addressing issues around misconceptions and awareness of the profession for different age groups to ensure healthcare career promotion is appropriate for each group and the use of effective sources of influence.

#### P.25 Second year health and social care students experiences of interprofessional education in the university setting

##### Catriona Doyle; Alison Power

###### Faculty of Health, Education and Society, University of Northampton, Northampton, UK

####### **Correspondence:** Catriona Doyle

**Background:** This study was based on a University of Northampton (UoN) project within the Undergraduate and Postgraduate Research Bursaries at Northampton (URB@N) to discover the interprofessional learning experiences of current second year healthcare students.

**Methods:** We based our research on the Readiness for Interprofessional Learning Scale (RIPLS) Questionnaire and adapted it to gather specific qualitative feedback on their experiences during first and second year at UoN.

**Results:** The qualitative feedback from the questionnaire responses did reinforce the view that students see the value of inter-professional training as part of the student’s learning experience. Some of the comments received included: *Understanding other professions roles is essential for effective teamwork and client centred care. An e-tivity to help explain roles would be useful.*

**Conclusions:** Overall the feedback received highlighted that students have an interest in getting to know their future work colleagues and in gaining a better understanding of the diverse roles of each profession which contributes to the overall holistic approach to client care. From this the faculty are going to devise a learning resource for current second years during their third year to prepare them for interprofessional working in the workplace.

#### P.26 Innovative use of visual technology for clinic based teaching

##### Claire Froggatt; Tracy Walker

###### Durham School of Podiatric Medicine at New College Durham, UK

####### **Correspondence:** Claire Froggatt

**Background:** The COVID-19 pandemic had a devastating effect on clinical teaching and clinical placement opportunities in all health professions but allowed us an opportunity to rethink traditional methods of clinical education and to consider how we could still offer a meaningful clinical teaching and placement experience for students using digital technology.

**Methods:** The initial aim was for a virtual clinical placement to be developed using head camera technology. This would allow students to undertake observational clinical teaching sessions regardless of the number of students, placement providers or future disruptions to normal provision. The head cameras chosen for use are fully voice activated, are robust and can be cleaned so eliminating issues with infection control.

**Results:** The chosen head camera technology is clear, can record and take photographs and has a built-in two-way audio. The real advantage to this system, however, is that the head camera can be linked to Microsoft TEAMs, a reliable platform that we and our students have been using throughout the pandemic. We are extremely fortunate to have several podiatry departments from local NHS trusts onboard to assist with the first stages of this project.

**Conclusions:** Although yet to incorporate this technology formally into the curriculum, we envisage that it will further expand placement opportunities for students by allowing them to remotely access other clinical professions such as vascular services and podiatric surgery teams. There is an acknowledgement however that no matter how good the virtual placement provision is, it is never intended to fully replace face to face patient encounters and hands-on practical work, but to enhance and expand current provision – future proofing the clinical educational experience.

